# Partial avulsion of the ulnaris lateralis and enthesiopathy of the lateral epicondyle of the humerus in a thoroughbred race horse

**DOI:** 10.1186/s13620-018-0120-6

**Published:** 2018-03-27

**Authors:** Neal Ashton

**Affiliations:** 1 Oakham veterinary hospital, Ashwell Road, Oakham, Rutland, LE15 7QH UK; 2 University of Nottingham Veterinary school, Sutton Bonnington Campus, Nottingham, LE12 5RD UK

## Abstract

**Background:**

There is relatively little literature on elbow disease in the horse. The only published reports on soft tissue conditions describe collateral ligament and biceps brachii injuries.

**Case presentation:**

A three-year old Thoroughbred gelding presented with a history of acute onset right forelimb lameness of less than one week duration, one month after commencing training in a National Hunt racing yard. Physical examination and peri-neural anaesthesia failed to localise the lesion. Lameness was localised to the distal humerus by nuclear scintigraphy and intra-articular anaesthesia. A partial avulsion of the tendinous origin of the ulnaris lateralis and enthesiopathy of the lateral humeral epicondyle was diagnosed on ultrasonography. Ultrasonographic findings were loss of longitudinal fibre pattern, a discrete hypo-echoic region within the ulnaris lateralis tendon of origin, and remodelling of the lateral epicondyle of the right humerus at the site of the origin of the ulnaris lateralis. No abnormalities were identified on radiography. The lameness did not resolve with rest, but was successfully treated with extra corporal shockwave therapy (ECSWT) and intra-lesional corticosteroid injections.

**Conclusion:**

This is the first report of partial avulsion of the ulnaris lateralis and enthesiopathy of the lateral humeral epicondyle in the horse. In contrast to other reported soft tissue conditions of the elbow, this horse had a successful return to work. This case highlights the value of ultrasonography in assessing peri-articular soft tissues, and the importance of pain relief as treatment in some soft tissue injuries.

## Background

Lameness originating from the elbow joint in horses is relatively uncommon, and there is currently limited evidence on diseases affecting the soft tissues of the elbow in the horse. Although there are reports of lateral collateral ligament injury [1], radial enthesiopathy of the elbow [2] and avulsion fracture of the ulnaris lateralis (personal communication [3]) in horses, avulsion of the ulnaris lateralis origin and enthesiopathy of the lateral epicondyle have not been reported. This case report describes the clinical presentation, diagnosis, treatment and successful outcome of a three-year old thoroughbred gelding with acute onset partial avulsion of the tendinous origin of the ulnaris lateralis, and enthesiopathy of the lateral epicondyle of the humerus.

## Case presentation

A three-year old Thoroughbred gelding presented with a history of acute onset right forelimb lameness of less than 1 week duration, 1 month after commencing training in a National Hunt racing yard. The horse was not yet in fast work. The trainer reported that he had been difficult to break in, but there had been no specific traumatic incident.

On presentation the gelding was 3/5 lame on the right fore in trot in a straight line [4]. On physical examination there were no focal sites of pain or swelling detected, a flexion test and extension test were negative on both forelimbs. An abaxial-sesamoid nerve block did not improve the lameness. The trainer elected not to investigate further at this time.

Following a period of 2 weeks box rest, the gelding re-presented at the hospital for further evaluation. There was no change in the clinical findings. The lameness was not altered by the following nerve blocks; palmar digital, abaxial-sesamoid, low 4-point, high 4-point and deep branch of the lateral palmar nerve. Intra synovial anaesthesia of the antebrachio-carpal and mid-carpal joints also did not alter the lameness. Medio-lateral and cranio-caudal radiographs of the shoulder and elbow joints were unremarkable. The trainer elected not to investigate further at this time.

Following a period of 6 weeks box rest there was no change in the lameness. There was atrophy of the right fore shoulder muscles, but no abnormalities or localising signs on physical examination. The horse was represented to the hospital for further investigation.

**Fig. 1:**
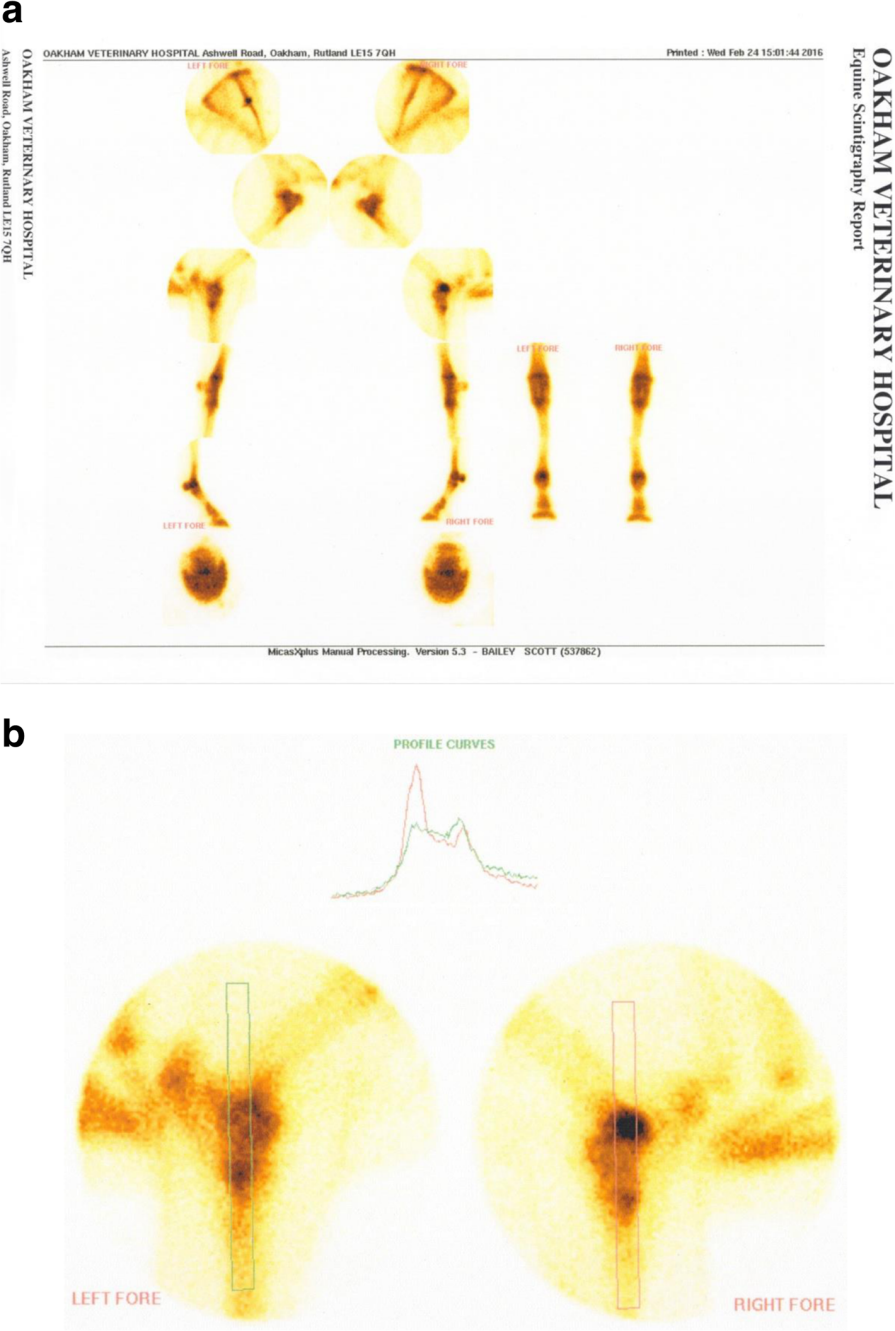
**a**, **b** Delayed phased nuclear scintigraphic images of a 3-year-old thoroughbred gelding with focal increased radiopharmaceutical uptake in the right fore distal humerus

Nuclear scintigraphy was performed: 7.5GBq of 99 m technetium-hydroxymethane diphosphonate (99mTc-HDP) was administered into the jugular vein. Images were obtained with a low-energy, general all-purpose collimator^1^ 2 hours after injection. There was focal increased radiopharmaceutical uptake in the region of the right fore distal humerus (Fig. 1a and b) Due to the absence of a cranial craniocaudal scintigraphic view, this could not be localized further on the basis of the scintigraphic findings.

Palpation, and focal pressure applied to the lateral epicondyle did not elicit a reproducible pain response from the horse. Flexion and extension tests were still unremarkable.

Intra-articular anaesthesia of the cubital joint was performed, 25 ml of mepivicaine^2^ was injected into the joint at the site of the palpable depression between the cranial aspect of the olecranon and the caudal aspect of the lateral epicondyle of the humerus. After 5 minutes the lameness was improved, and the horse was sound after 15 min.

**Fig. 2:**
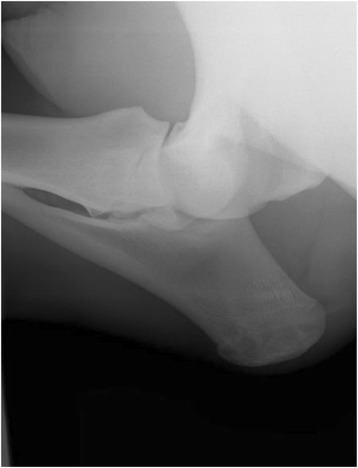
Mediolateral radiographic views of the elbow of a 3 year old thoroughbred gelding with lateral partial avulsion of the ulnaris lateralis and enthesiopathy of the lateral epicondyle of the humerus. No radiographic abnormalities were identified

**Fig. 3:**
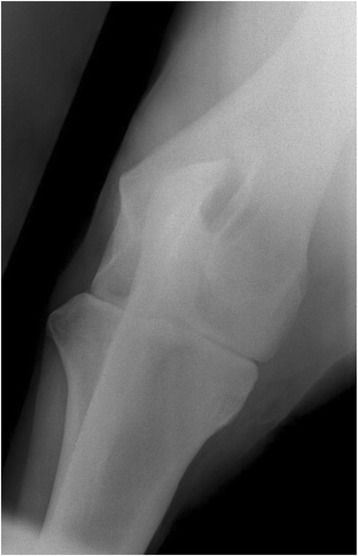
Craniocaudal radiographic views of the elbow of a 3 year old thoroughbred gelding with lateral partial avulsion of the ulnaris lateralis and enthesiopathy of the lateral epicondyle of the humerus. No radiographic abnormalities were identified

Repeat radiographs of the elbow joint (medio-lateral and cranio-caudal) revealed no abnormalities (Figs. 2 and 3).

**Fig. 4:**
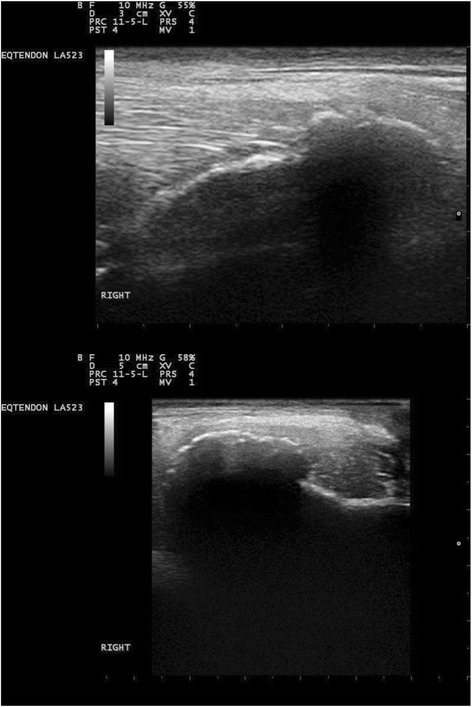
Ultrasonographic image of the lateral aspect of the elbow of a 3 year old thoroughbred gelding. Longitudinal and transverse images of the lateral epicondyle of the right elbow. There is an irregular bone surface and remodelling of the lateral epicondyle of the right humerus at the site of the origin of the ulnaris lateralis tendon. The fibre pattern of the ulnaris lateralis tendon was disrupted on the longitudinal view, with a discrete hypo-echoic region at the site of origin onto the lateral epicondyle

**Fig. 5:**
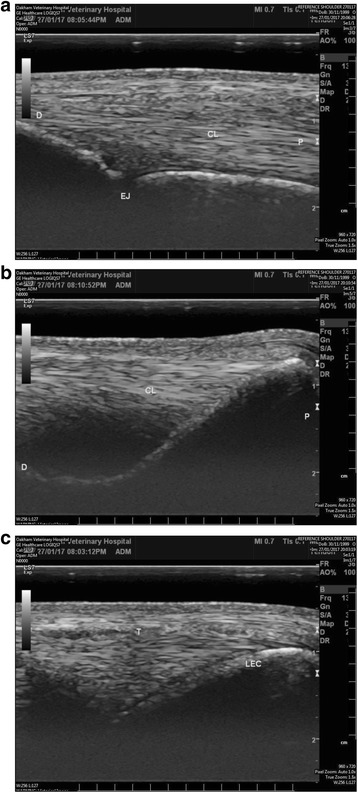
Reference ultrasonographic images. **a** Longitudinal ultrasonogram of the origin of the ulnaris lateralis on the lateral epicondyle of the humerus. T-ulnaris lateralis tendon; LEC-lateral epicondyle of the humerus. **b** Transverse ultrasonogram of the origin of the ulnaris lateralis on the lateral epicondyle of the humerus, T-Ulnaris lateralis tendon; LEC lateral epicondyle of the humerus. **c** Longitudinal ultrasonogram of the origin of the lateral collateral ligament of the elbow. CL lateral collateral ligament of the elbow; P- proximal; D-distal

Ultrasound examination of the left and right elbow joints, were performed using a 10mHz linear transducer.^3^ Transverse and longitudinal views of the lateral aspect of the elbow showed an irregular bone surface and remodelling of the lateral epicondyle of the right humerus at the site of the origin of the ulnaris lateralis tendon [5]. The fibre pattern of the ulnaris lateralis tendon was disrupted on the longitudinal view, with a discrete hypo-echoic region at the site of origin onto the lateral epicondyle (Fig. 4). A reference ultrasound image (obtained from a different 3yo thoroughbred horse) of the ulnaris lateralis origin on the lateral epicondyle is shown in fig. 5a and b, in addition a reference image of the origin of the collateral ligament of the elbow is shown in Fig. 5c.

### Diagnosis

The presence of bone modelling and the ultrasonographic changes within the ulnaris lateralis tendon were considered to be consistent with a diagnosis of a partial avulsion of the origin of the ulnaris lateralis and enthesiopathy of the lateral epicondyle of the humerus.

### Treatment

Following discussion with the owner, a period of paddock rest was undertaken, after 6 weeks the gelding was improved to 1/5 lame.

A further five-month period of paddock rest was undertaken.

At re-presentation there was no further change in the clinical signs (1/5 lame), or ultrasonographic findings. Following aseptic preparation, the ulnaris lateralis tendon of origin was injected with 10 mg of triamcinalone (Adcortyl), the lateral epicondyle of the humerus was then treated with extracorporal shock wave therapy^4^ at 3.0 Bar with a 15 mm transmitter for 3000 pulses. The trainer reported that he was sound 3 days later and he was started in work. The lameness recurred after 6 weeks, the same treatments were repeated, and 3 days later the horse was sound and able to resume work. A third treatment was carried out 5 weeks later, although the horse remained sound and the shoulder muscle atrophy had resolved.

There was no recurrence of the lameness. There have been no further instances of lameness in the subsequent 18 months, and the horse has raced with no further related issues.

## Discussion and conclusions

Lameness attributable to the elbow region is relatively uncommon in the horse. Soft tissue injuries to the collateral ligament and enthesiopathy of the biceps brachii tendon have been described [1, 2], but avulsion of the ulnaris lateralis and remodeling of the lateral epicondyle of the humerus, have not. There is a single description of avulsion of the ulnaris lateralis, as a personal communication [3], of a case with radiographic changes.

The diagnosis in this case was based on increased radionucleotide uptake in the region of the humeral epicondyle, a positive response to local anaesthesia of the elbow joint and ultrasonographic findings of bone remodelling and changes in the ulnaris lateralis tendon.

The elbow is a large joint therefore radiography in the horse is not sensitive to focal subtle changes. It is possible that further radiographic projections would have revealed the new bone formation identified ultrasonographically.

Intra-articular analgesia of the elbow joint significantly improved the lameness in this case, despite the lesion being extra-articular. It is most likely that the local anaesthetic diffuses out of the volumous caudoproximal joint space to eliminate pain from the lateral epicondyle. Had local anaesthesia been performed earlier in the investigation, imaging of the elbow region would have been indicated and the diagnosis could have been reached without the cost of scintigraphy. However, as this condition has not been previously reported, scintigraphy was valuable in ruling out more common injuries with similar presentation, such as stress fractures or other bony abnormalities, and in identifying the distal epicondyle of the humerus as the site of pathology.

ECSWT has been shown to be beneficial in providing pain relief for insertional tendinopathies and desmopathies in man [4, 6] and is widely used in the horse. The usual frequency of treatment in man is once weekly; in this case the interval was 6 weeks; therefore the corticosteroid injection may have contributed more significantly to the duration of pain relief. However it is also possible that the condition was self-limiting and would have resolved spontaneously when training was resumed.

In man lateral elbow tendinopathy (LET) is a common condition causing pain on the lateral aspect of the distal humerus. Although there are similarities with this case, the soft tissue structures affected differ; it is caused by a stressed common extensor origin lateral epicondyle complex of the effected elbow [7]. In cases of LET the diagnosis is primarily based on the patient’s description of the problem, focal pain, the results of physical examination and exacerbation tests, such as gripping tests. The most common imaging modalities for confirming the diagnosis in LET are ultrasonography and MRI [7], Nuclear scintigraphy has also been reported to be valuable [8]. Ultrasonography is considered a reliable imaging technique for LET [5], findings are similar to this case with hypoechogenicity of the soft tissues involved and bone changes of the lateral epicondyle [5, 7]. There are many conservative treatments proposed for LET including ECST [4, 6]. Corticosteroid injections are thought to be useful in the acute stage but potentially detrimental in the chronic stage [9], as chronic LET appears to have no inflammatory component [10]. There are several surgical options for treatment of recalcitrant cases of LET, these are variously supported by reported evidence, they include denervation of the lateral humeral epicondyle [11] and percutaneous release under local anaesthesia [12]. In man, the tendinous origins at the lateral epicondyle are accessible arthroscopically from the elbow joint, subequently the most recently described surgical options are arthroscopic release techniques [13].

The case described did not resolve with rest and free exercise for a total of over 6 months. The combination of intra-lesional corticosteroids and extra corporal shockwave therapy (ECSWT) were used to provide pain relief and facilitate a return to exercise. This resulted in strengthening of the shoulder muscles as observed by the resolution of shoulder muscle atrophy. It is most likely that the improved muscle strength was able to compensate for the injured structure and thus resolve the lameness. Given the response to intra-articular analgesia, arthroscopic evaluation of the elbow joint could have been considered an option in this case. The diagnosis was based on increased radionucleotide uptake in the region of the humeral epicondyle on scintigraphy and the ultrasonographic findings of bone remodelling and changes in the ulnaris lateralis tendon.

Partial avulsion of the ulnaris lateralis and enthesiopathy of the lateral epicondyle of the humerus should be considered as a differential diagnosis for horses presenting with acute onset forelimb lameness, with no localising signs, that does not block to the distal limb, or resolve with short-term periods of rest. In contrast to other reported soft tissue injuries in the elbow, this horse successfully returned to exercise beyond his previous level. This case highlights the value of ultrasonography in assessing peri-articular soft tissues, and the importance of pain relief to facilitate exercise and allow strengthening of muscles to compensate for some cases of soft tissue injury.

## Abbreviations

ECSWT: Extra coporal shockwave therapy
LET: Lateral elbow tendinopathy
MRI: Magnetic resonance imaging

## Footnotes

1 General Electric, Bartec Medical Imaging Systems, Camberley, Surrey, England.

2 Intra-Epicaine® 20 mg/ml, Dechra Veterinary Products, Shrewsbury, England.

3 LogiqS7 Expert, BCF Technology, Strathclyde Business park, Scotland.

4 Masterpuls®, MP200Vet, Storz medical, 8274 Tägerwilen, Switzerland
